# Larvicidal effects of selected medicinal plant extracts against *Anopheles arabiensis, Anopheles stephensi,* and *Aedes aegypti*

**DOI:** 10.1186/s41182-025-00879-2

**Published:** 2025-12-31

**Authors:** Negesse Gebissa, Ketema Tolossa, Araya Gebresilassie, Esayas Aklilu, Daniel Bisrat, Bersissa Kumsa, Sisay Dugassa

**Affiliations:** 1 Aklilu Lemma Institute of Health Research, Addis Ababa University, PO Box 1176, Addis Ababa, Ethiopia; 2 Department of Zoological Sciences, College of Natural and Computational Sciences, Addis Ababa University, PO Box 1176, Addis Ababa, Ethiopia; 3 College of Health Science, Addis Ababa University, PO Box 1176, Addis Ababa, Ethiopia; 4 College of Veterinary Medicine and Agriculture, Addis Ababa University, PO Box 1176, Bishoftu, Ethiopia

**Keywords:** Mosquitoes, Larvae, Medicinal plants, Malaria, Arboviral diseases, Bioinsecticides

## Abstract

**Background:**

The emergence of resistance to synthetic (chemical) insecticides along with their harmful effects on human health, non-target organisms and the environment necessitates the development of new complementary bioinsecticides that are effective, environmentally friendly, biodegradable and target-specific. This study was undertaken to evaluate larvicidal activities of 80% methanol and n-hexane extracts of four plants that are traditionally used by communities against mosquitoes*.*

**Methods:**

The dried plant parts of *Ocimum lamiifolium*, *Amaranthus hybridus, Premna schimperi*, and *Lepidium sativum* were extracted with 80% methanol and n-hexane solvents. Bioinsecticidal activities of these extracts were evaluated under laboratory condition in the range of 62.5–2000 ppm against late 3rd to early 4th instar larvae of *An. arabiensis, An. stephensi* and *Ae. aegypti* mosquitoes. Larval mortality was observed after 24 h of exposure. The mortality data were subjected to probit analysis to determine LC_50_ and LC_90_ values.

**Results:**

In the concentration ranges of 62.5–2000 ppm, the LC_50_ and LC_90_ values of the most potent n-hexane extracts tested plants; *Ocimum lamiifolium* against *An. arabiensis, An. stephensi* and *Ae. aegypti* with a general ranges 666.07 to 1278.22, and 1920.82 to 2139.91*,* and *Amaranthus hybridus* against *An. stephensi* and *Ae. aegypti* 412 to 1426.03 and 736.150 to 1222.62*, Lepidium sativum* and *Premna schimperi* against *An. arabiensis* exhibited 100% larvicidal activity with LC_50_ and LC_90_ values ranges 713.25 to 1278.22, and 636.76 to 988.90, respectively. All the n-hexane extracts showed larvicidal activities.

**Conclusions:**

The n-hexane crude extracts of the tested plants have the potential to be used as bioinsecticides against larvae of *An. arabiensis, An. stephensi* and *Ae. aegypti*. Therefore, it is necessary to undertake studies that focus on bioassay-guided isolation, purification and structural elucidation of active compound (s) from the most active n-hexane fractions of the tested plants to develop a product that may complement the current existing vector control tools.

## Background

Mosquito-borne diseases (MBDs) are increasingly prevalent due to the resultant impact of global change with significant health and economic impacts worldwide [1]. *Anopheles* and *Aedes* mosquitoes are among the most important groups of arthropods with medical significance that transmit several important parasitic and arboviral diseases [2]. Malaria, a life-threatening disease, is primarily transmitted to humans through the bites of infected female *Anopheles* mosquitoes, including *An. arabiensis* and *An. stephensi* [3]. *An. arabiensis* is also a major malaria vector within the *An. gambiae* species complex in sub-Saharan Africa and surrounding islands [4].

Arboviruses continue to generate significant health and economic burdens for people living in endemic regions, such as East Africa, including Ethiopia [5]. These viruses (dengue, Zika, chikungunya, and yellow fever virus) are transmitted mainly by *Aedes* mosquitoes [6]. Arbovirus infections are a global public health threat accounting for approximately 73% of the total emerging and re-emerging human infections, where the burden is worsened in sub-Saharan Africa, including Ethiopia [7]. Malaria, caused by protozoans and arboviral diseases caused by viruses can lead to high mortality and morbidity in tropical and sub-tropical regions [8]. The spread of these mosquito vectors in different parts of East African Region including Ethiopia has become a serious concern for malaria and arboviral diseases prevention and elimination strategies [9]. Global malaria cases and deaths have been significantly reduced following the scaling up of long-lasting insecticidal nets (LLINs) and indoor residual spraying (IRS) [10]. However, widespread use of synthetic insecticides in controlling mosquito vectors has resulted in the persistence and accumulation of non-biodegradable chemicals in the ecosystem, development of resistance to insecticides in vectors, and toxic effects in non-target organisms, including humans [11].

As evidenced in recent studies from different parts of Ethiopia, all the three mosquito species have shown resistance to insecticides belonging to the four chemical classes (such as organochlorines, organophosphates, carbamates, and pyrethroids) and approved for IRS and ITNs [12]. There is also evidence of changes in biting and resting behavior of the main malaria vectors as a result of selective pressures by the widespread and long-term use of bed nets and indoor residual spraying [13]. The emergence of synthetic insecticides resistance necessitates an urgent need to develop natural product-based mosquito control methods that are biodegradable, economical, effective and less toxic to non-target organisms, including edible aquatic animals, humans and the environment [14]. In this regard, plant extracts with bioinsecticidal potential are recognized as complementary and/or alternative plant-based products to synthetic insecticides in mosquito control programs due to their ovicidal, larvicidal, adulticidal and repellence properties that may enhance the discovery of botanical-based products that are safe, biodegradable, and target-specific natural agents [15].

Widespread resistance to synthetic (chemical) insecticides used to control disease-carrying mosquitoes poses a global health threat. The use of plants in the production of bioinsecticides is vital under these circumstances.

Therefore, the aim of this study was to assess the effectiveness of plant extracts that are repeatedly used traditionally as a larval poison for mosquitoes that transmit diseases in Ethiopia, following the standard WHO bioassay test methods and procedures using the chemical insecticide Temephos as positive control.

## Materials and methods

### Selection and collection of plant species

The plant species that were evaluated for insecticidal properties were collected following the ethno-taxonomic approach by searching targeted plant families from the literature in the manner discussed by Martin [16]. The information on bioinsecticidal plants were collected from literature reports. Different ethno-botanical publications by different researchers were reviewed. Appropriate data collection format was prepared to tabulate scientific, family and local names of species. To select the most relevant and promising plant species, the candidate test plant species were listed by taking the fidelity level (Fidelity level is a statistical tool to measure the potentiality of a specific plant for treating a specific disease [16] into consideration. Here, the citation frequency of medicinal plants (at least in 3 reviewed articles) and the availability of important and adequate information of each of the medicinal plants in the reviewed documents were taken into consideration [17]. Accordingly, the candidate plant species were initially compiled from literatures and followed by field sampling of the test plants.

Four plant species, namely, *Ocimum lamiifolium*, *Lepidium sativum* L., *Amaranthus hybridus* L. and *Premna schimperi* Engl., were selected, and collected based on their traditional use as against insects*.* The plant materials both (voucher specimens, and materials for extraction and testing) were collected during field trips at the sites in the months of August and November, 2024, respectively, from Oromia Regional State, Jimma zone, Asendabo district and Southwest Ethiopia Regional State, Kaffa Zone, Bonga district, Ethiopia.

### Voucher specimen collection and identification

The plant specimens were identified by the plant taxonomy specialist, Mr. Melaku Wondafirash and deposited at National Herbarium, Department of Plant Biology and Biodiversity Management, College of Natural and Computational Sciences, Addis Ababa University, Ethiopia for future references. All the field data were properly recorded, summarized and presented in Table 1.

**Table 1:** Field data and ethno-botanical information of plants used in this study

**S.N**	Species name	Family name	Plant parts	Collection Place	Local name	Habitat	GPS coordinates	Altitude	References
1	*Ocimum lamiifolium* Hochst. Ex. Walp (LG-01)	Lamiaceae	Aerial parts	Asendabo	Damakase (AO)	Home	(07^0^46′15.155″N) (037^0^14′03.585″E)	1,761 m	[17–19]
2	*Lepidium sativum* L.(LG-10)	Lamiaceae	Seed	Asendabo	Feto (AO)	Stead	(07^0^43′20.34″N) (36^0^18′09.79″E)	1,772 m	[20–22]
3	*Amaranthus hybridus* L. (LG-06)	Amaranthaceae	Aerial parts	Bonga	Asangira (AO)	Forest	(07^0^43′20.34″N) (36^0^18′09.79″E)	1,772 m	[23–25]
4	*Premna schimperi* Engl. (LG-04)	Lamiaceae	Leaves	Asendabo	Urgessa (AO)	Wild	(07^0^46′05.846″N) (037^0^14′02.226″E)	1,757 m	[26–28]

### Bulk sample collection and processing

The plant of *Ocimum lamiifolium* (aerial parts), *Lepidium sativum* (seeds), *Premna schimperi* (leaves) and *Amaranthus hybridus* (aerial parts) were collected and kept separately in plastic bags, tied with rubber bands and brought to Aklilu Lemma Institute of Health Research (ALIHR) laboratory, Addis Ababa University, Ethiopia. The collected plant materials were air-dried under shade at room temperature (25 ± 2 °C) for 7 days. The dried plant materials were separately powdered using electrical grinder and sieved to get 1-mm particle sizes to improve the subsequent extraction by rendering the sample more homogenous, increasing the surface area and facilitating the penetration of solvent into cells. The powdered plant materials were placed in air-tight plastic bags, labeled and stored in dark box at room temperature until used for crude extract preparation and bioassays.

### Preparation of crude extracts

Hundred grams of each finely powdered plant material was separately soaked in a clean 500 ml flask with 80% methanol and n-hexane solvent systems for extraction at a ratio of 1:5 (w/v) at room temperature for 3 days with continuous stirring at 120 rpm using shaker to ensure complete extraction. At the end of extraction, the micelle of each solvent system was separated from marc by filtration using four layered cloths. Afterward, the crude extracts were centrifuged by HERMLE (Z 383 K) apparatus at 1500 rpm for 5 min for clarification of the filtered sample extracts. Subsequently, the clear filtrates of extracts of both solvent systems were concentrated using rotary evaporator (*DREHSCHIEBR-VAKUUMPUMPE* ROTARY VANE VAUUM PUMP) under reduced pressure at 40 °C, while the remaining aqueous portions were collected separately in amber glass bottles, and then evaporated to dryness using an oven set at a temperature of 40 °C. The dried plant extracts were weighed, the percentage yields determined and stored in amber-colored vials at 4 °C until the stock solutions were prepared and used for bioassays.

### Rearing of mosquitoes

Larvae of three mosquito vectors were reared at the insectary of the Aklilu Lemma Institute of Health Research, AAU, Ethiopia. The eggs of *Anopheles arabiensis*, *Anopheles stephensi*, and *Aedes aegypti* were placed in a tray with tap water under laboratory conditions. After 24 h of incubation, the eggs hatched into first instar larvae. An appropriate amount of nutrient, comprised of sterilized yeast powder and dog biscuit in a 1:1 ratio, was added to promote the growth of the larvae. The late third-to-early fourth instar larvae were utilized in the study. The larvae colonies were maintained under standardized conditions in a separate room in the insectary at 25 ± 2 °C and 60% ± 10% relative humidity, following a 12:12 light–dark photoperiod cycle to ensure the reliability and reproducibility of the data.

### Larvicidal bioassay

The bioinsecticide bioassays against laboratory-reared mosquitoes were carried out following the standard procedures of WHO larvicidal test method [26]. Twenty ml of 1% (w/v) stock solutions of each extract was prepared by dissolving 200 mg extracts in 20 ml of 5% dimethyl sulfoxide and twofold serial dilutions of the plant extracts test concentrations (2000, 1000, 500, 250,125 and 62.5 ppm) were prepared. The n-hexane extracts of the four selected plant species, namely, *Ocimum lamiifolium, Lepidium sativum, Premna schimperi* and *Amaranthus hybridus,* were evaluated against late 3rd to early 4th instar larvae of *An. arabiensis, An. stephensi* and *Ae. aegypti* mosquitoes and then subjected to a dose dependent bioassay to determine LC_50_ and LC_90_ values.

For the bioassay, stock solutions of each extract test concentrations of the plant extracts were serially diluted with 5% dimethyl sulfoxide as described in the WHO larvae bioassay protocol [26]. The mixtures were gently stirred to ensure a homogeneous test solution and kept at ambient temperature. Determination of the desired lower concentrations were prepared by serial dilutions based on the dilution principle following the formula C_1_V_1_ = C_2_V_2_ as described [29].

One hundred fifty (150) larvae of a given species were required to conduct a single dose–response tests; of these, 100 were exposed to the concentration being tested (4 replicates each of 25 mosquitos’ larvae) plus 25 larvae in the negative control and 25 larvae in the positive control groups. Each assay was repeated three times. At the end of the 24 h, the number of dead larvae was recorded and the percentage mortality was calculated using the formula [30].

The percentage of mortality = (number of dead larvae/total number of larvae used)*100In the first phase of bioassay, the bioinsecticidal activity of 80% methanol and n-hexane crude extracts of frequently used candidate test plants, namely, *O. lamiifolium, L. sativum, A. hybridus* and *P. schimperi,* were screened at 2000 ppm concentration. The mosquitoes’ larvae were exposed to 2000 ppm of test concentration and a control to find out the activity of the materials under test. Based on the preliminary screening, four levels of classifications were used to determine efficacy level of extracts: strong mortality > 80%; moderate mortality 80–60%; weak mortality 60–40%; little or no activity mortality < 40%. Hence, extracts yielding the mortality of larvae > 40% were selected and subjected to dose–response bioassay. Whereas, none larval mortalities were observed in the corresponding negative control, while the corresponding positive control exhibited 100% mortalities of the respective mosquito larvae during the preliminary screening.

### Data analysis

Results were expressed as the mean percent mortality with standard deviations. One-way analysis of variance (ANOVA) using SPSS for windows (version 26) was used to test differences in mean larval mortality rates between crude extracts. Larvicidal activity was considered to be significantly different when 95% confidence limit levels failed to overlap or if *P* value < 0.05. Comparisons between mean percent mortality rates of larvae that were treated with crude extracts, negative and positive controls were made using Turkey’s post hoc testing. The tests were conducted with 4 replicates per concentration, and the experiment was repeated 3 times and then the mean mortality rates of 12 replicates were taken to determine the percent mortality after 24 h of exposure using Turkey’s post hoc HSD test. The average larval mortality data were subjected to general linear probit model analysis for calculating LC_50_, LC_90_ and other statistics at 95% confidence limits of upper confidence limit and lower confidence limit, and chi-square values were calculated.

## Results

### Plant species collected for the larvicidal tests

The dried crude extracts were weighed and their percentage yield also calculated. The scientific names, solvents used for extraction and yield of crude extracts of the selected plants used for bioinsecticide bioassays are summarized in Table 2.

**Table 2:** Percentage yield of 80% methanol and n-hexane crude extracts of targeted plant species evaluated against larvae of *An. arabiensis*, *An. stephensi,* and *Ae. aegypti*

S.N	Plant species	Wt (%)	
		80% methanol	n-hexane
1	*Ocimum lamiifolium* Hochst. ex. Walp	5.31	4.7
2	*Lepidium sativum* L	11.3	8.1
3	*Amaranthus hybridus* L	9.7	2.9
4	*Premna schimperi*	13.3	4.8

A total of four extracts from four different medicinal plants were tested for bioinsecticide activities against *An. arabiensis, An. stephensi* and *Ae*. *aegypti*. The bioinsecticidal activity of the candidate test plant species of 80% methanol and n-hexane crude extracts were evaluated. From these, the n-hexane crude extracts of *O. lamiifolium, L. sativum, A. hybridus* and *P. schimperi* were selected and subjected to dose–response bioassays at concentrations of 2000, 1000, 500, 250, 125 and 62.5 ppm (Table 3) and yielding larvae mortality of > 40% after 24 h during the preliminary screening, while all 80% methanol crude extract treated larvae exhibited mortalities under 40% classification range in the first phase of bioassay or during the screening.

**Table 3:** Mean percentage mortality against larvae of *An. arabiensis, An. stephensi* and *Ae. aegypti* of various concentrations of n-hexane crude extracts from the test plants after 24 h of exposure. All values are in triplicate; (*) implies no mortality

Plant Species	Mosquito species	Conc. (PPM)	Mean %	MD	SD	95% CI		Sig
						LB	UP	
*Lepidium sativum* L	*An. arabiensis*	5% DMSO	100.0	1.00	5.810	118.04	81.96	1.000
		2000	100.0*	− 100.00*	5.810	− 118.04	− 81.96	.000
		1000	90.7*	− 90.7*	5.810	− 108.71	− 72.63	.000
		500	41.3*	− 41.3*	5.810	− 59.37	− 23.29	.000
		250	26.7*	− 26.7*	5.810	− 44.71	− 8.63	.005
		125	13.7*	− 13.7	5.810	− 31.71	4.37	.547
		62.5	1.67*	− 1.7	5.810	− 19.71	16.37	1.000
	*Ae. aegypti*	2000	97.3*	− 97.3*	3.540	− 113.54	− 79.96	.000
		1000	89.0*	− 89.0*	3.540	− 103.29	− 69.71	.000
		500	62.3*	− 62.3*	3.540	− 61.79	− 28.21	.000
		250	13.5*	− 13.5	3.540	− 30.29	3.29	.999
		125	3.5 − *	− 3.5	3.540	− 20.29	13.29	1.000
		62.5	− *	.0	3.540	− 16.79	16.79	1.000
*O. lamiifolium* Hochst. ex. Walp	*An. arabiensis*	2000	100.0*	− 100.0*	4.672	− 106.17	− 77.16	.000
		1000	93.0*	− 93.0*	4.672	− 99.17	− 70.16	.000
		500	17.0*	− 17.0*	4.672	− 23.17	5.84	.585
		250	10.3*	− 10.3*	4.672	− 16.51	12.51	1.000
		125	6.0*	− 6.0	4.672	− 12.17	16.84	1.000
		62.5	2.3*	− 2.6	4.672	− 106.17	− 77.16	1.000
	*An. stephensi*	2000	99.7*	− 99.7*	.691	− 101.81	− 97.52	.000
		1000	7.0*	− 7.0*	.691	− 9.15	− 4.85	.000
		500	.7*	− 0.7	.691	− 2.81	1.48	.978
		250	.4*	− .4	.691	− 2.48	1.81	1.000
		125	− *	.0	.691	− 2.15	2.15	1.000
		62.5	− *	.0	.691	− 2.15	2.15	1.000
	*Ae. aegypti*	2000	100.0*	− 100.0*	.744	− 102.31	− 97.69	.000
		1000	91.7*	− 91.7*	.744	− 93.31	− 88.69	.000
		500	8.0*	− 8.0*	.744	− 10.31	− 5.69	.000
		250	− *	.0	.744	− 2.31	2.31	1.000
		125	− *	.0	.744	− 2.31	2.31	1.000
		62.5	− *	.0	.744	− 2.31	2.31	1.000
*Amaranthus hybridus* L								
	*An. arabiensis*	2000	31.3*	− 31.3*	1.298	− 35.36	− 27.30	.000
		1000	3.0*	− 3.0	1.298	− 7.03	1.03	.299
		500	.7*	− .7	1.298	− 4.70	3.36	1.000
		250	− *	.0	1.298	− 4.03	4.03	1.000
		125	− *	.0	1.298	− 4.03	4.03	1.000
		62.5	− *	.0	1.298	− 4.03	4.03	1.000
	*An. stephensi*	2000	100.0*	− 100.0*	.734	− 109.11	− 90.89	.000
		1000	62.3*	− 62.3*	.734	− 71.44	− 53.22	.000
		500	5.0*	− 5.0	.734	− 14.11	4.11	.685
		250	.4*	− .4	.734	− 9.44	8.78	1.000
		125	− *	.0	.734	− 9.11	9.11	1.000
		62.5	− *	.0	.734	− 9.11	9.11	1.000
	*Ae. aegypti*	2000	100.0*	− 100.0*	1.449	− 104.50	− 95.50	.000
		1000	83.3*	− 83.3*	1.449	− 87.83	− 78.83	.000
		500	29.3*	− 29.3*	1.449	− 33.83	− 24.83	.000
		250	2.5*	− 2.5	1.449	− 6.50	2.50	.864
		125	− *	.0	1.449	− 4.50	4.50	1.000
		62.5	− *	.0	1.449	− 4.50	4.50	1.000
*Premna schimperi* Engl	*An. arabiensis*	2000	100.0*	− 100.0*	1.999	− 106.21	− 93.79	.000
		1000	82.0*	− 82.0*	1.999	− 88.21	− 75.79	.000
		500	12.3*	− 12.3*	1.999	− 18.54	− 6.13	.967
		250	− *	.0	1.999	− 6.21	6.21	1.000
		125	− *	.0	1.999	− 6.21	6.21	1.000
		62.5	− *	.0	1.999	− 6.21	6.21	1.000
	*Ae. aegypti*	2000	70.3*	− 70.3	1.441	− 74.81	− 65.86	.000
		1000	56.3*	− 56.3	1.441	− 60.81	− 51.86	.000
		500	1.3*	− 1.3	1.441	− 5.81	3.14	.983
		250	− *	.0	1.441	− 4.47	4.47	1.000
		125	− *	.0	1.441	− 4.47	4.47	1.000
		62.5	− *	.0	1.441	− 4.47	4.47	1.000
		Temephos 0.25	− 100.000	− 100.0	5.810	− 118.04	− 81.96	.000

There were statistically significant differences in mean percentage mortality of the respective mosquito larvae among different concentrations of the n-hexane plant extracts at ≥ 500 ppm compared with negative control (*P* < 0.05) (Table 3). Statistical results (one-way ANOVA) have showed significantly high larvicidal activity (100% mortalities) of n-hexane solvent crude extracts (*P* < 0.00) after 24 h exposure to four extracts of the test plants at concentrations of 2000 ppm, against *An. arabiensis* mosquito larvae

**Fig. 1:**
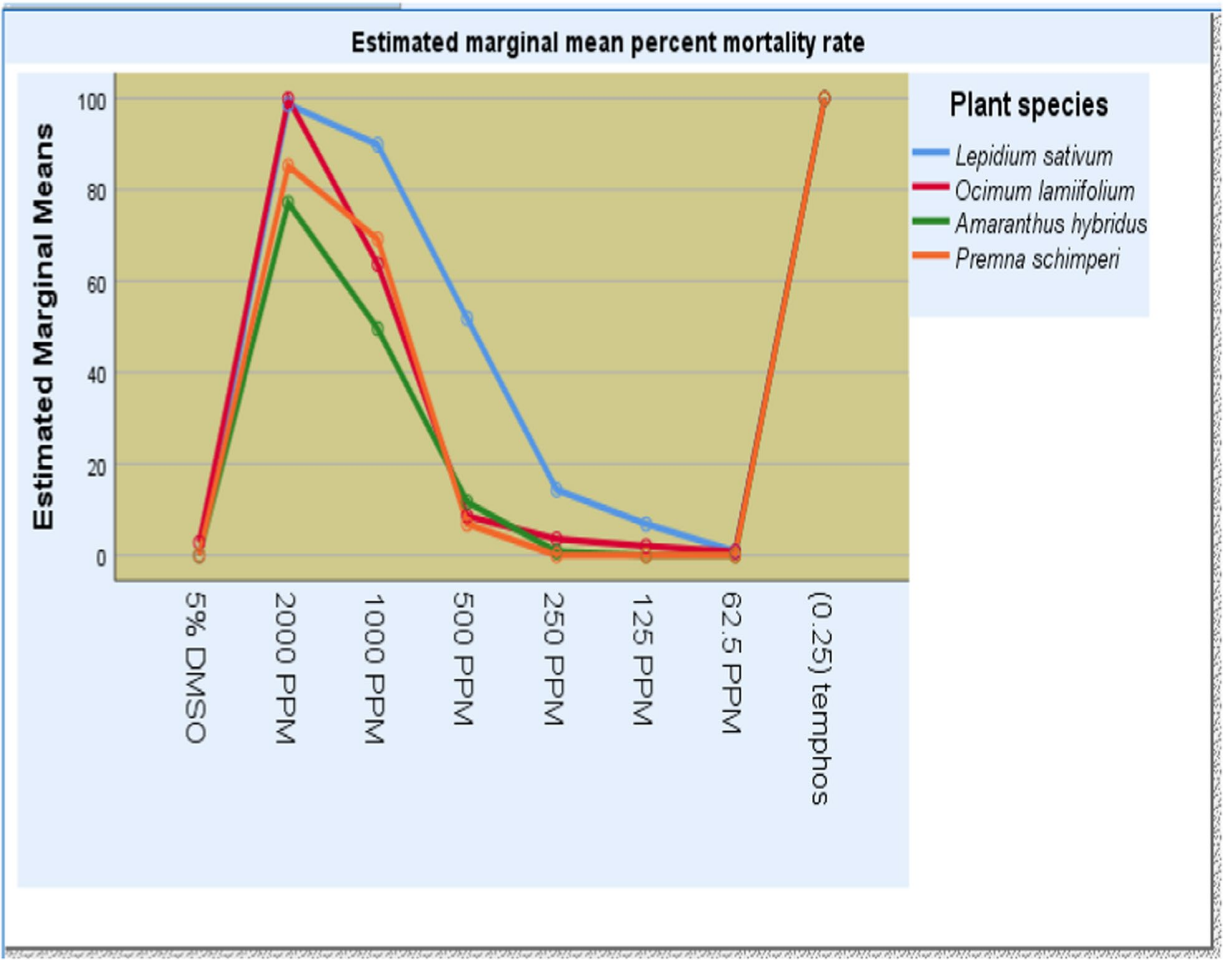
Bioinsecticide activity of various concentrations of n-hexane crude extracts from the test plants: *Ocimum lamiifolium*, *Lepidium sativum*, *Amaranthus hybridus*, and *Premna schimperi* against *Anopheles arabiensis*, *Anopheles stephensi*, and *Aedes aegypti*

The results of larval mortality were obtained from bioassays of the n-hexane crude extracts of *O. lamiifolium, L. sativum, A. hybridus* and *P. schimperi* against *An. arabiensis, An. stephensi* and *Ae*. *aegypti* at different concentrations after 24 h exposure periods are presented in Table 3 and Fig. 1.

All n-hexane crude extracts of test plants showed low, moderate, and high bioinsecticidal activities between 62.5 and 2000 ppm treatments against the late 3rd to early 4th instar larvae of the respective mosquito larvae. Within the same exposure period, larval mortality was not recorded at lower concentrations (≤ 250 ppm) of extracts and the negative control, while the standard (temephos) achieved 100% of larval mortality.

The present study also showed that n-hexane crude extracts of *Lepidium sativum* and *Ocimum lamiifolium* had pronounced bioinsecticide activity followed by *Amaranthus hybridus* and *Premna schimperi*. Significantly higher bioinsecticide activity (*P* < 0.00) was exerted by *Ocimum lamiifolium* at a concentration ≥ 500 ppm. Around 100% mortalities against late 3rd to early 4th instar larvae of *An. arabiensis*, *An. stephensi* and *Ae. aegypti* by *Ocimum lamiifolium* and *Amaranthus hybridus*, and *An. arabiensis* by *Lepidium sativum* and *Premna schimperi* crude extracts were recorded, respectively, after 24 h of exposure at a concentration of 2000 ppm the same effect as 0.25 ppm temephos (*P* > 0.05). However, there were no statistically significant difference (*P* > 0.05) in the bioinsecticidal potential of all n-hexane extracts at the lower concentrations (*P* < 500 ppm). The mortality effect of the test plant extracts against the test mosquitoes’ larvae were dose dependent. No activity was observed in the negative control, while the positive control exhibited 100% larvae mortality (Table 3). Larvae mortality increased with increased concentrations of the tested crude extracts (Fig. 1).

Table 3 indicates one-way ANOVA (statistical) results that had showed significantly higher bioinsecticidal activities (*P* < 0.00) of the respective mosquito larvae after 24 h of exposure to *Ocimum lamiifolium, Lepidium sativum* and *Amaranthus hybridus* n-hexane crude extracts at concentrations ≥ 500 ppm.

### Determination of LC_50_ and LC_90_ of the crude extracts

The lethal effects of four different test plant species crude extracts against larvae of the targeted vector mosquitos were subjected to probit analysis. The LC_50_ and LC_90_ values of the n-hexane crude extracts are shown in Table 4.

**Table 4:** Lethal concentration of n-hexane crude extracts against larvae of *An. arabiensis, An. stephensi,* and *Ae. aegypti* after 24 h of exposure (ppm) *According to probit analysis (Finney, 1971) % Mortality = mean ± SD

Plant extracts	Mosquito species	X^2^	LC_50_	95% CI		LC_90_	95% CI	
				LB	UB		LB	UB
*Lepidium sativum*	*An. arabiensis* ^(b,c)^	5.7	545.36	451.61	669.48	982.160	822.99	1260.60
	*Ae. Aegypti* ^(b,c)^	6.2	642.24	423.37	755.90	1073.86	955.99	1260.06
*Ocimum lamiifolium*	*An. arabiensis* ^(b,c)^	13.8	843.70	633.50	1388.21	1386.79	1016.89	2452.24
	*An. stephensi* (^b,c^)	19.8	1278.22	940.42	4537.28	1717.44	1309.95	9365.05
	*Ae. aegypti* ^(b,c)^	.004	713.25	622.76	815.92	988.90	858.00	1255.86
*Amaranthus hybridus*	*An. arabiensis*^(b,c)^	.408	2333.96	1985.3	3246.98	3225.87	2623.69	5155.50
	*An. stephensi*^(b,c)^	.963	874.78	744.23	1031.01	1240.27	1092.87	1545.77
	*Ae. aegypti* ^(b,c)^	.167	636.76	536.86	755.90	1046.01	915.37	1260.06
*Premna schimperi*	*An. arabiensis*^(b,c)^	.050	736.15	633.92	854.78	1073.76	955.99	1271.54
	*Ae. aegypti* ^(b,c)^	6.4	1209.30	987.32	1525.25	2230.20	1538.14	7879.38

a) *LC_50_ Lethal concentration that kills 50% of the exposed larvae, LC_90_ Lethal concentration that kills 90% of the exposed larvae, X^2^ chi-square, LB lower boundary limit, UB Upper boundary limit

b) A heterogeneity factor is used for split file Botanic crude extracts = *Ocimum lamiifolium*, exposed mosquito species = *An. arabiensis* mosquito larvae

c) A heterogeneity factor is used for split file Botanic crude extracts = *Ocimum lamiifolium*, exposed mosquito species = *An. stephensi* mosquito larvae

## Discussion

Due to environmental concerns and the development of insect resistance to synthetic insecticides, the recent trend is to evaluate plants to obtain extracts that are safe for non-target animals and do not pose any residue problem but are still able to suppress vector populations. In view of this, the present study tested the bioinsecticidal activities of 80% methanol and n-hexane crude extracts of *Ocimum lamiifolium, Lepidium sativum, Amaranthus hybridus* and *Premna schimperi* plants against *An. arabiensis, An. stephensi,* and *Ae. aegypti*. In this study, all 80% methanol crude extracts showed little/no bioinsecticidal activities on the target malaria and viral infections vectors, while n-hexane crude extracts of the test plants achieved lower, moderate, and high bioinsecticidal activities. This suggests the presence of more non-polar solvent–soluble phytochemicals in the tested plant species and parts, namely, *Ocimum lamiifolium, Lepidium sativum, Amaranthus hybridus* and *Premna schimperi,* which are responsible for the observed bioactivities of these plants against larvae of *An. arabiensis, An. stephensi* and *Ae. aegypti* mosquitoes.

The rationale for the use of solvents with different polarity such as water (*P* = 1.00), methanol (*P* = 0.762), and n-hexane (*P* = 0.009) and methanol gradient with water such as 80% methanol was because different organic solvents and their combination with water show difference in dissolving the bioactive plant secondary metabolites present in the plant materials. The use of different solvents with various polarities is necessary, because different solvents can significantly affect the potency of extracted plant compounds [31]. This has also been shown by [32] in oak gall extraction and [33] in neem plant extraction, whereby a converse relationship between extract effectiveness and solvent polarity was observed. It had also reported that the extraction of more bioactive components from *Acorus calms* (Acoraceae) that had more lethal effect on adult mosquitoes with certain solvents than others [34].

The current findings of the bioassay revealed that the n-hexane extract was more effective than the 80% methanol extracts. This is consistent with earlier reports [35] that showed decline in mortality of mosquitoes with increasing solvent polarity of a mosquitocidal plant extract. These authors showed that water extract of *Zanthoxylum heitzii* (Rutaceae) produced low adult mortalities, whereas its ethyl acetate and n-hexane extracts produced higher mortalities on *Anopheles gambiae*. From this, it is clear that the bioactive components responsible for the lethal effect on mosquitoes are extracted in greater amount and potency with certain solvents only and not with all. The current finding of bioassay of polar (80% methanol) and non-polar (n-hexane) plant extracts against *An. arabiensis, An. stephensi* and *Ae*. *aegypti* mosquito larvae was in line with the findings of earlier studies. The present study demonstrated that the bioinsecticidal activities of n-hexane extracts were much higher compared to the 80% methanol extracts implies that the potency of the active constituents in the crude extracts might have been masked by non-polar and major/minor constituents.

All the n-hexane extracts showed bioinsecticidal activities against larvae of *An. arabiensis* and *Ae. aegypti* mosquitos. Among the tested extracts, the high bioinsecticidal activity shown by n-hexane extract of *Ocimum lamiifolium* against the larvae of the three mosquito vectors in Ethiopia is not surprising, since it has been reported as a multipurpose traditional medicine. Due to its pharmacological effects, this plant has been widely used traditionally for the treatment of headaches, coughs, diarrhea, constipation, warts, and kidney damage [18]. These properties come from the secondary metabolite components that are abundant in *Ocimum* plants, such as steroids, tannins, alkaloids, flavonoids, and phenolics [36]. In addition, the abundant components of essential oils make *Ocimum* a plant that can fight the growth of organisms [37]. *Ocimum lamiifolium* has many pharmacological properties that are the reason why they are well-known, praised, and widely used as home remedies [37].

The most widely used mosquito repellent plant reported in Ethiopia is *Allium sativum* L. followed by *Lepidium sativum* L. and *Capparis tomentosa* Lam*.* [20, 21]. Abebe et al. [21] reported that bioinsecticides were made locally to kill insects by spraying all over the walls of the house. It is plausible to assume that it is the phytochemicals contained in such plants that are responsible for the bioinsecticidal activities against both the larvae and adult stages of mosquitoes.

The findings of the present study also indicated that n-hexane crude extract of *Amaranthus hybridus* showed statistically significant bioinsecticidal activity (*P* < 0.000) against late third-to-early fourth instars larvae of *An. arabiensis, An. stephensi* and *Ae. aegypti* at concentrations of 2000, ≥ 1000, and ≥ 500 ppm, respectively, after 24 h exposure. The result of this finding is consistent with the research, [23] which showed the larvicidal activity of the same plant extract and their Cu nanoparticles (extract materials sized, from 1 to 100 nm in diameter) against *Culex* and *anopheles*’ larvae. The highest mortality was found at 50 mg/L with the lethal dose or lethal concentration (LC) of 50% and 90% mortality calculated to be 0.861 and 0.995, respectively. The mortality is attributed to the presence of phytochemicals in the leaves of the plant extract. The current work also can be compared to the work of [24]. Similarly, the finding of [38] where ethanol leaf extract of *D. stramonium* was found to cause 70.56% mortality against 3rd instar *An. gambiae* larvae at 1000 ppm in Eritrea, though the concentrations are different. The petroleum ether extract of *Datura stramonium* also showed strong efficacy against the 4th instar larvae *Ae. aegypti* 100% mortality after 24 h of exposure [39]. The difference in bioinsecticidal activity of the current finding could be due to the difference in species and parts of plants, concentrations, extraction solvent, and season of collection and agro ecology of the plants.***

The biological activities of the phytochemicals that include alkaloids, terpenoids, steroids, phenols, saponins and tannins extracted from several tropical plants have been receiving the attention of many researchers as potential sources of mosquitocides and for treatment of various vector-borne diseases [31, 40]. The crude extracts that caused high mortality to mosquitos’ larvae contained plant secondary metabolites, such as alkaloids, phenols, flavonoids, saponins, and cardiac glycosides with insecticidal effects [23, 41]. Therefore, the high bioinsecticidal potencies of n-hexane crude extracts of the four plants were the basis for considering further screening studies on the different solvent partitioned fractions against the three targeted mosquito vectors.

The ethyl acetate extract of *L. sativum* damaged the midgut of *Cx. pipiens* larvae, interfering with development and survival. A similar result was obtained when *Ae. aegypti* (Linnaeus in Hasselquist) (Diptera: Culicidae) larvae were treated with *Schinus terebinthifolius* Raddi (Sapindales: Anacardiaceae) extract, resulting in disorganization and damage in the midgut in comparison with the control [19]. Different plant derived larvicides caused deleterious effects in the larvae midgut, including vacuolization, cell hypertrophy, damage to microvilli, cell lysis, degeneration of epithelial cells, disruption of osmoregulation, and damage to the gastric caeca [42–44]. The remarkable larvicidal and ovicidal activities of *L. sativum* ethyl acetate extract might be due to the presence of phenol, which is known to possess promising insecticidal activity. Several compounds from different plants such as phenolics, steroids, alkaloids, essential oils, and terpenoids have been reported as promising potential insecticides [31], either in pure or crude extract form. The larvicidal effects of plant secondary metabolites vary based on plant species, parts used, geographical region, mosquito species, extraction methodology, and the polarity of the solvents used during extraction [45]. In the present study, all 80% methanol crude extracts caused lower bioinsecticidal activities, only n-hexane solvents crude extracts were subjected to dose response bioassay to detect the lethal concentrations. The n-hexane extract of *Lepidium sativum* caused the highest bioinsecticidal activities against the late 3rd to early 4th instar larvae of *An. arabiensis* and *Ae. aegypti* with the lowest LC_50_ of 545.360 ppm and LC_90_ of 982.160, *Amaranthus hybridus* showed the relatively highest LC_50_ and LC_90_ (LC_50_ = 2857.24 ppm, LC_90_ = 6683.15 ppm) and, 642.244 and 1073.864, respectively. Similarly, the highest bioinsecticidal activities against *An. stephensi* larvae were exhibited by n-hexane extract of *Amaranthus hybridus* with the lowest LC_50_ of 874.78 ppm and LC_90_ of 1240.27 ppm values. This difference could be related to differences in environmental factors. In his review, [46] indicated that plant secondary metabolite accumulation is strongly dependent on a variety of environmental factors, such as light, temperature, soil water, soil fertility and salinity, and for most plants, a change in an individual factor may alter the content of secondary metabolites even if other factors remain constant.

The n-hexane extract of *Amaranthus hybridus* demonstrated the most significant bioinsecticidal activity against *An. stephensi* larvae, with an LC_50_ value of 874.78 ppm and an LC_90_ value of 1240.27 ppm. Similarly, [25] reported that, petroleum ether extract of leaf powder *Amaranthus hybridus* resulted in the LC_50_ of 409.87 ppm against 3rd instar larvae of *Culex* species after 24 h of exposure. The variations in bioinsecticidal activities may be attributed to the differences in susceptibility of the test species and the type of solvent used. The differences observed in the current study's bioinsecticidal activity may stem from these factors. Consequently, it is recommended to pursue bioassay-guided fractionation, isolation, and characterization of the bioactive constituents from the crude extracts of the four plants utilized in this research.

## Conclusion

This study demonstrated that the n-hexane crude extracts of *Ocimum lamiifolium*, *Lepidium sativum*, and *Premna schimperi* possess significant bioinsecticidal activity against the late 3rd to early 4th instar larvae of *Anopheles arabiensis* and *Aedes aegypti* mosquitoes. Furthermore, the n-hexane crude extract of *Ocimum lamiifolium* showed even greater bioinsecticidal activity against larvae of the *An. stephensi* mosquito. The complete mortality of larvae from *Amaranthus hybridus* crude extract at 2000 ppm against both *An. stephensi* and *Ae. aegypti* larvae indicates the potential of this plant for the development of botanical bioinsecticides targeting the mosquito vectors studied. However, additional research is necessary to assess their effectiveness in field conditions. Overall, these four plants may be considered as viable candidates for the development of effective, safe, biodegradable, and cost-effective botanical bioinsecticides for vector control, potentially enhancing resistance management against the larvae of mosquito vectors in Ethiopia and other regions. Therefore, further bioassay-guided phytochemical studies on the promising plants and their extracts to formulate and develop plant-based products as mosquito vector control strategy are highly recommended. Accordingly, further bioassay-guided phytochemical investigations on these promising plants extracts are strongly recommended to formulate and develop plant-based products for mosquito vector control strategies.

### Limitations

The main limitations of this study include:

- The effectiveness of the extracts was not assessed and validated in field settings or against all developmental stages of the targeted vector.
- In addition, due to financial constraints, bioassay-guided phytochemical studies were not undertaken to identify and characterize the bioactive compounds from the selected plant extracts.

## Data Availability

The data sets used and/or analyzed during the current study may be obtained from the corresponding author on reasonable request.

## Abbreviations

ANOVA: Analysis of variance
IRS: Indoor residual insecticide spraying
LLINs: Long-lasting insecticide-treated mosquito nets
SPSS: Statistical Package for the Social Sciences
LC: Lethal concentration
